# Experimental Study of the Dynamic Characteristics of a New Antidrainage Subgrade Structure for High-Speed Railways in Diatomaceous Earth Areas

**DOI:** 10.3390/ma15020532

**Published:** 2022-01-11

**Authors:** Qian Su, Zhixing Deng, Xun Wang, Wenyi Jia, Yunbin Niu

**Affiliations:** Department of Civil Engineering, Southwest Jiaotong University, Chengdu 610031, China; suqian.sju@outlook.com (Q.S.); 2020210063@my.swjtu.edu.cn (Z.D.); Jwy@my.swjtu.edu.cn (W.J.); yunbinniu@my.swjtu.edu.cn (Y.N.)

**Keywords:** high-speed railway, diatomaceous earth, engineering properties, new antidrainage roadbed structure, dynamic response

## Abstract

The experience needed to carry out engineering and construction in diatomaceous earth areas is currently lacking. This project studies the new Hang Shaotai high-speed railway passing through a diatomaceous earth area in Shengzhou, Zhejiang Province, and analyzes the hydrological and mechanical properties of diatomaceous earth on the basis of a field survey and laboratory. Moreover, a new antidrainage subgrade structure was proposed to address the rainy local environment, and field excitation tests were performed to verify the antidrainage performance and stability of the new subgrade structure. Finally, the dynamic characteristics and deformation of the diatomaceous earth roadbed were examined. The hydrophysical properties of diatomaceous earth in the area are extremely poor, and the disintegration resistance index ranges from 3.1% to 9.0%. The antidrainage subgrade structure has good water resistance and stability under dynamic loading while submerged in water. After 700,000 loading cycles, the dynamic stress and vibration acceleration of the surface of the subgrade bed stabilized at approximately 6.37 kPa and 0.94 m/s^2^, respectively. When the number of excitations reached 2 million, the settlement of the diatomaceous earth foundation was 0.08 mm, and there was basically negligible postwork settlement of the diatomaceous earth foundation. These results provide new insights for engineering construction in diatomaceous earth areas.

## 1. Introduction

Diatomite (diatomaceous earth) is a rock (soil) made of diatom shells deposited over a long period. Diatom shells are the cell walls of the decayed internal material of diatoms, and the main component is hydrated silica, which has a hard texture. The presence of diatoms makes the internal porosity of diatomite (soil) high [1]; in addition, diatomite (soil) also exhibits low density, a low permeability coefficient, a strong structure, good adsorption, and easy disintegration in the presence of water [2,3,4].

Diatomaceous earth is widely used in various fields, such as environmental protection, building materials, and the chemical industry because of its strong adsorption and small skeleton [5,6,7,8,9,10], but relatively little research has been conducted on its mechanical and engineering properties. The main studies thus far in terms of mechanical properties are as follows. Day et al. tested diatomaceous soils from the southern region of California in the United States. They found that the soils had high water content and low dry density, and that they exhibited high compressibility and low shear resistance [11]. Ovalle et al. observed the behavior of in situ natural diatomaceous earth under compression, shear, and cyclic loading through microscopy and found that the mechanical behavior of diatomaceous earth did not follow traditional geomechanical laws [12]. Perisic investigated the mechanical behavior of diatomaceous soils in the Gulf of Mejillones, northern Chile, and found that the diatomaceous soils in this area have a large friction angle, a high initial void ratio, high natural water content, and very low density [13]. R. Shiwakoti et al. found that the compressibility of the soil and the angle of internal friction increased as the content of the diatomaceous earth increased [14]. Tae-Hyuk Kwon et al. studied the geotechnical properties of diatomaceous earth in the eastern waters of Korea and concluded that the microporous structure of diatomaceous earth increases its specific surface area, leading to a decrease in the specific gravity of the earth, which, in turn, leads to the high compressibility and plasticity of diatomaceous earth [15]. The existing engineering examples include only bridge pilings and road slopes, and there is a lack of experience needed to study and analyze the engineering properties of diatomaceous earth for the construction of large-scale projects. Wang analyzed a variety of the material properties of diatomaceous earth at the Barker Creek Bridge through in-house and in situ testing methods and found that diatomaceous earth had “nontextbook” engineering properties, with a very high natural moisture content near or at the liquid limit, and a high overconsolidation ratio [16]. Zhang Yongshuang et al. investigated the physical and mechanical properties and engineering geological characteristics of diatomaceous earth from highway slopes in the Tengchong area of Yunnan and found that the diatomaceous soils in the region had high porosity, high water absorption, high structural properties, and high plasticity, and that they exhibited the disintegration characteristics of expansive soils [17]. Guo Changbao conducted shear tests on remolded diatomaceous earth and found that disturbed diatomaceous earth was strain-softened soil [18]. As the density of high-speed railroad networks gradually increases, the construction of high-speed railroads will inevitably pass through special soil areas. It is important to carry out relevant research for the soils along the line to ensure the smooth development of the engineering construction.

The new Hang Shaotai Railway could not completely bypass the diatomite (diatomaceous earth) layer when selecting the line. This was because of the restrictions for the location of the new Shengzhou Xinchang Station. There were still five work sites containing diatomite (diatomaceous earth) in the stratum, within a total of approximately 3.2 km of the subgrade length. According to preliminary geological survey tests, diatomite (diatomaceous earth) should be considered a special geotechnical soil, but the current specification does not include it in the survey specification. There is little experience in conducting relevant geological surveys, so the physical and mechanical properties of diatomite need to be analyzed and studied in depth to guide the design and construction of high-speed railways built in diatomite areas.

In summary, to ensure the smooth development of the Hang Shaotai high-speed railroad construction in the diatomaceous earth area of Shengzhou, the engineering properties of diatomaceous earth and the service performance of the diatomaceous earth subgrade structure were studied by integrating various test methods. Firstly, the hydraulic and mechanical properties of the diatomaceous earth were analyzed on the basis of a field survey and indoor tests. Then, a new type of drainage prevention subgrade structure was proposed for the first time for the diatomaceous earth area, and field excitation tests were carried out to simulate the most unfavorable conditions, coupling dynamic load and water immersion, to analyze the drainage prevention performance and durability of the new drainage prevention layer. Finally, the long-term stability, the deformation mechanism, and the control methods of the diatomaceous earth subgrade structures were studied in order to provide references and new insights for the construction of related projects in diatomaceous earth areas.

## 2. Project Overview

**Figure 1:**
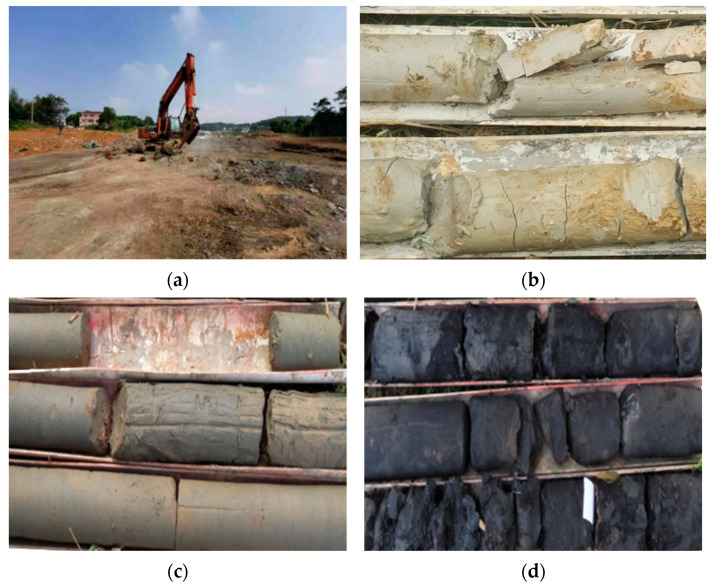
Test section site and three types of diatomaceous earth: (**a**) test site; (**b**) white diatomaceous earth; (**c**) blue diatomaceous earth; and (**d**) black diatomaceous earth.

As shown in Figure 1a, in order to study the engineering properties of diatomaceous earth, the natural foundation graben section of diatomaceous earth from mileage DK86 + 130 to DK86 + 160 was selected as the test site. The lithology of the strata in the test site is mainly Quaternary Holocene alluvium (Q4al + pl) chalky clay, Upper Tertiary Pliocene (N2) white (blue and black) diatomaceous earth, and porous basalt.

The diatomaceous earth in the test section is mainly composed of the remains of diatoms, formed in the basaltic and multiphase fluvial–lacustrine sedimentary layers of the Tertiary multiphase eruption. In the investigated section, the diatomite color varies significantly along the depth direction of the foundation, with white, blue, and black diatomite distributed from top to bottom, and the layer thickness varies from 4 to 90 m, as shown in Figure 1b–d.

## 3. Hydrophysical and Physical Properties of Diatomaceous Earth

**Figure 2:**
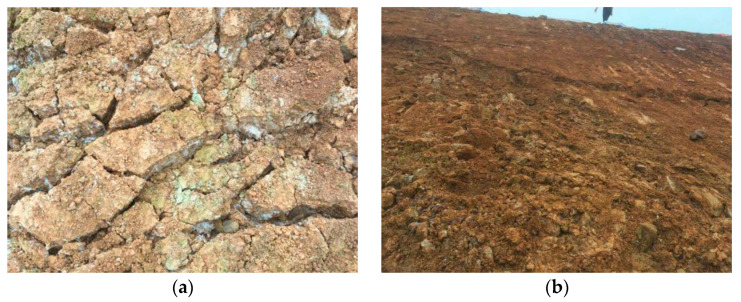
Disintegration damage under the action of wet and dry cycles: (**a**) disintegration damage; and (**b**) severe soil damage.

Prior studies have shown that diatomaceous earth has the undesirable property of deteriorating when exposed to water [19,20,21,22,23], and the climate in Shengzhou exhibits alternating wet and dry cycles, so outdoor observation tests were carried out first. A portion of the excavated diatomaceous earth layer was selected as the test section and was covered with colored cloth only for initial protection, and its condition was observed after one month. After a period of wet and dry cycling, the diatomaceous earth layer cracked, the shallow layer of the slope broke up and lost its original structure, the diatomaceous earth disintegrated into blocks, and the depths of the fissures were between 0.5 and 0.8 m, as shown in Figure 2.

To solve the problem of diatomaceous earth damage under the action of wet and dry cycles, the basic physical properties of diatomaceous earth in the laboratory was analyzed first. Then, through observations of the hydrophysical properties of diatomaceous earth and disintegration tests, the changes in the diatomaceous earth after exposure to water were grasped. Finally, a disintegration and deterioration analysis of the diatomaceous earth was carried out to understand the mechanism for disintegration and deterioration, which is expected to provide a reference for the design and construction of diatomaceous earth road bases.

### 3.1. Basic Physical Properties of Diatomaceous Earth

**Figure 3:**
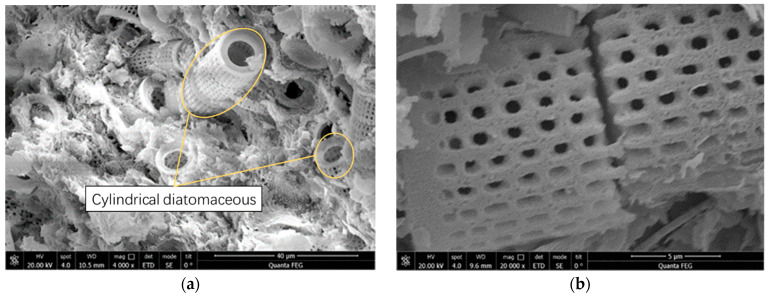
Microstructures of diatomaceous earth determined by scanning electron microscopy: (**a**) white diatomite (4000×); and (**b**) multilevel pores of diatomite.

The three-phase relationship of the soil and its natural structure has a great influence on the engineering properties of the soil, and understanding the physical properties and physical state of the soil is important for understanding the engineering properties of the soil [24,25]. Therefore, representative samples were selected for indoor experiments near the Hang Shaotai railroad project. Scanning electron microscopy experiments were carried out on 19 diatomite samples, and the results are shown in Figure 3. A total of 134 diatomite samples were tested for conventional indexes, and the results are shown in Table 1. Scanning electron microscope tests were conducted using an environmental scanning electron microscope, and the magnification range was taken at 2000~50,000 times during the tests. The conventional index test was conducted according to the Geotechnical Test Procedure for Railway Engineering [26]. The drying method was used to test the moisture content of the text block, the oven temperature was 105 ℃~110 ℃, the drying time was more than 8 h, and the weighing accuracy was 0.01 g. The ring knife method was used to test the density of the text block; the inner diameter of the ring knife was 6–8 cm, the height was 2–3 cm, the wall thickness was 1.52 mm, and the balance weighing accuracy was 0.01 g. From these results, the following relationships can be inferred:

1. The diatomaceous earth contained diatom remains of various shapes that were mainly cylindrical. Because of the dense distribution of extremely small round pores (pore size < 0.2 μm) all along the walls of the diatom remains and the mixed accumulation of the diatom remains, the diatomaceous earth exhibited extremely high porosity and very low density;
2. The water content of diatomaceous earth was very high, and the water content of the black diatomaceous earth ranged from 27.5 to 102.2%, with an average water content of 68.02%, which showed that the natural diatomaceous earth exhibited high humidity;
3. The diatomaceous earth had very large pore ratios, with an average pore ratio between 1.47 and 1.85, which is an important reason for the high-water content of diatomaceous earth;
4. The results of the tests carried out on samples taken at different depths show that the natural density, the water content, and the pore ratio of the diatomaceous earth tended to decrease at shallow depths and increase at deeper depths. This is related to the fact that the diatomaceous earth located closest to the surface is most affected by changes in the external environment, both wet and dry.

### 3.2. Observations of the Hydrophysical Properties of Diatomaceous Earth

**Figure 4:**
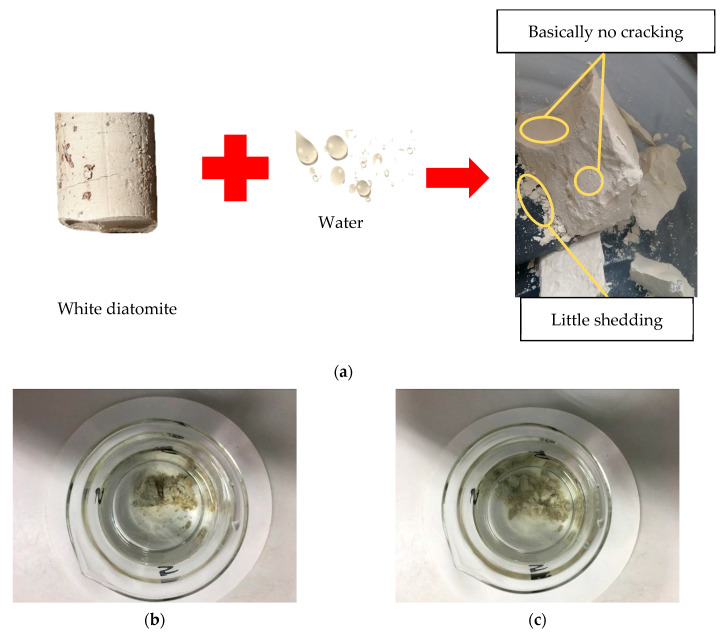
Hydrophysical property diagram of diatomaceous earth: (**a**) natural diatomaceous earth in contact with water; (**b**) initial stage of water disintegration of dry diatomaceous earth; and (**c**) end stage of water disintegration of dry diatomaceous earth.

To grasp the changes occurring in diatomaceous earth when it is exposed to water, an indoor observation test of the hydrophysical properties of diatomaceous earth was carried out. The diatomaceous earth samples collected on-site were divided into two groups to observe the reactions of different samples when exposed to water under dry and natural conditions. It was seen that:

1. Natural diatomaceous earth did not change significantly when immersed in water and was more stable than dry diatomaceous earth, as shown in Figure 4a;
2. Because of its low specific gravity, dry diatomaceous earth floated on the surface of the water for a few seconds, and then absorbed the water and sank, rapidly disintegrating into a fine, scaly, powdered form, as shown in Figure 4b,c;
3. When natural diatomaceous earth was dried in the shade, fissures developed. When the lower part was immersed in water, the water was preferentially transported through the fissures and transferred to the upper part, which was not immersed in water. The natural diatomaceous earth broke up in blocks and flaps after exposure to the sun.

### 3.3. Disintegration Characteristics of Diatomaceous Earth

Five specimens (average mass of 15.88 g) without macroscopic fissures were selected for indoor disintegration studies to test the time of the disintegration, the total disintegration, and the disintegration resistance of the specimen mass. The test interval was 10–20 s, and the weighing accuracy was 0.01 g.

The disintegration resistance index (%) was used to evaluate the disintegration characteristics of individual specimens; it is the ratio of the residual mass of the specimen after disintegration to the total mass of the specimen:

(1) $$I=\frac{M_{r}}{M_{t}}\times 100\%$$

where *I* is the resistance to the disintegration index (%); *M_r_* is the mass of the residual sample (g); and *M_t_* is the total mass of the sample (g).

**Figure 5:**
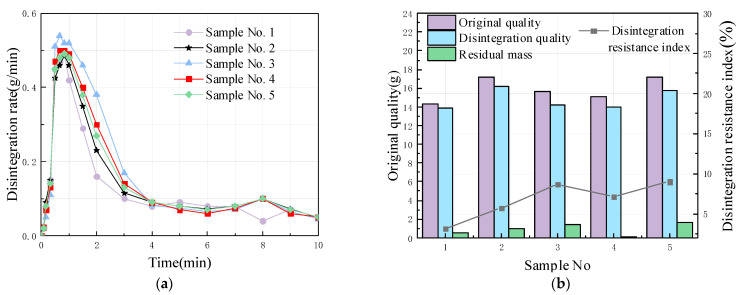
Test results of sample disintegration characteristics (average mass of 15.88 g): (**a**) comparison of sample disintegration rates; and (**b**) comparison of sample resistances to disintegration index.

On the basis of the results of the observation tests, the following relationships can be inferred:

1. As shown in Figure 5a, during the first 30 s after the specimen was placed in water, the disintegration rate was low as the water gradually entered the pores of the specimen during this time, and there was still some air enclosed in the pores by water. As the test proceeded, more water entered the pore space, and the gas enclosed in the pore space was further compressed, resulting in the tensile stress of the gas on the soil. When the strengths of certain weak parts of the specimen were less than the tensile stress, fractures were produced. The specimen disintegrated rapidly at 1 to 2 min into the test, after which the disintegration rate decreased and stabilized until the end of the test;
2. As shown in Figure 5b, the maximum value of the disintegration resistance index for the five specimens was 9.0%, with almost complete disintegration. The disintegration resistance index ranged from 3.1% to 9.0%, with very weak disintegration resistance and strong disintegration.

The results of the combined laboratory and outdoor observations show that the hydrophysical properties of diatomaceous earth are extremely poor and are prone to both disintegration and damage under alternating wet and dry effects. In actual engineering construction, subjecting the lower diatomaceous earth foundation to alternating wet and dry effects should be avoided as much as possible. Therefore, the antidrainage performance of the new Hangtai railroad roadbed needs to be strengthened in order to avoid the occurrence of the abovementioned diseases.

## 4. Antidrainage Measures and Excitation Test Methods

### 4.1. New Antidrainage Subgrade Structure

**Figure 6:**
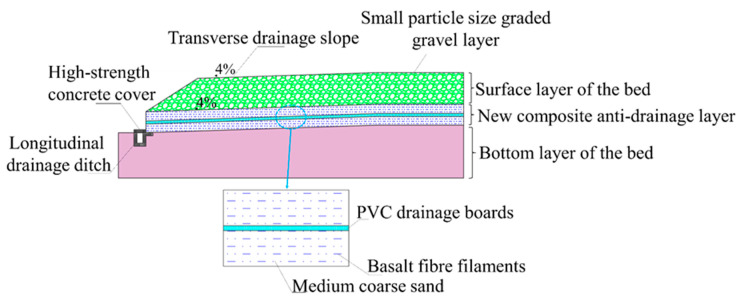
Diagram of the new antidrainage subgrade structure in the diatomaceous earth area.

As shown in Figure 6, in view of the wet and rainy environment in Shengzhou, a new antidrainage subgrade structure was designed for the diatomaceous earth area in the test section to enhance the antidrainage performance of the subgrade. The new antidrainage subgrade structure was designed to include two parts: the material for the antidrainage layer, and the new antidrainage subgrade structure. The material for the drainage prevention layer was a new drainage prevention layer composite material, which was made of medium-coarse sand as a skeleton, reinforced by basalt fiber filaments, and interspersed with PVC drainage boards. The new antidrainage road base structure consisted of a 4% transverse drainage slope from the center to both sides of the road base, the surface layer of the bed, the new composite antidrainage layer, the bottom layer of the bed, and the drainage ditch (top cover).

### 4.2. Simulation of Water Immersion Environments

**Figure 7:**
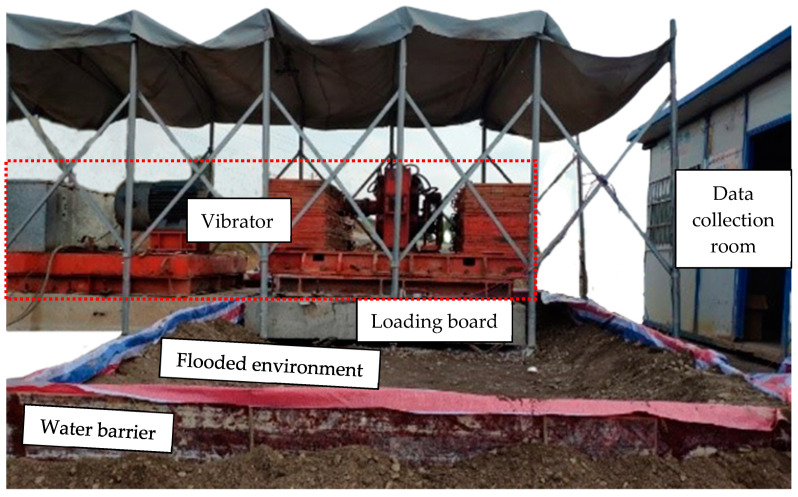
Excitation test equipment and simulation of water immersion conditions.

To test the drainage performance and stability of the new drainage prevention subgrade bed structure, it was necessary to use the water barrier to simulate flooding conditions. Therefore, a water barrier was constructed on the surface of the bed in the test section. The water barrier was 7 m wide, 3.5 m long, and 0.3 m high along the cross-sectional direction of the line, with a double layer of colored fabric around it, and a plugging treatment inside to avoid water leakage during construction. Water had to be injected into the water barrier at all times during the test to keep the surface of the bed covered with water, as shown in Figure 7.

### 4.3. Excitation Test Program

**Figure 8:**
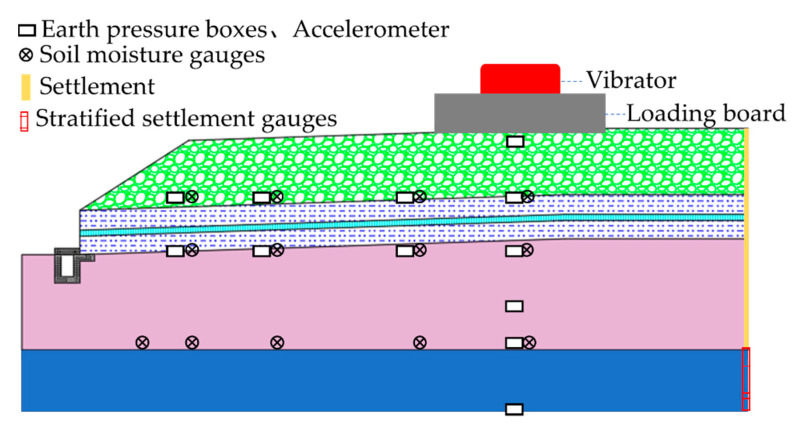
Sensor arrangement diagram.

The DK86 + 150 section was selected as the test section for the field excitation test, and the dynamic response during the excitation test was monitored by burying the earth pressure boxes and accelerometers on site. At the same time, settlement plates, stratified settlement gauges, and soil moisture gauges were installed to monitor the cumulative settlement of the subgrade and foundation surfaces and the changes in the internal water content, as shown in Figure 8.

### 4.4. Excitation Test Equipment and Loading Parameters

This test used in situ excitation test equipment developed by the Department of Road and Railway Engineering of Southwest Jiaotong University; this was a high-speed railway subgrade bed in situ dynamic test system (referred to as DTS-1), which mainly consisted of a vibration frame, an exciter, a transmission system, a circulation cooling system, and an electrical control system, as shown in Figure 7. The main technical parameters of the field excitation test are as follows.

#### 4.4.1. Stress Amplitude

According to the design code for high-speed railways [27], the design dynamic stress amplitude of the subgrade was calculated according to the following formula:

(2) $$\sigma_{j}=0.26\times p$$

(3) Δσ=0.26×p×αv

(4) $$\sigma_{d}=0.26\times p\times (1+\alpha v)=\sigma_{j}+\Delta \sigma$$

where $\sigma_{j}$ is the static stress generated by the axle weight of the train on the roadbed surface (kPa); p is the axle load of the train (200 kN); ∆σ is the additional dynamic stress caused by the train movement (kPa); αv is the dynamic stress growth factor; and $\sigma_{d}$ is the design stress amplitude of the roadbed surface (kPa).

**Figure 9:**
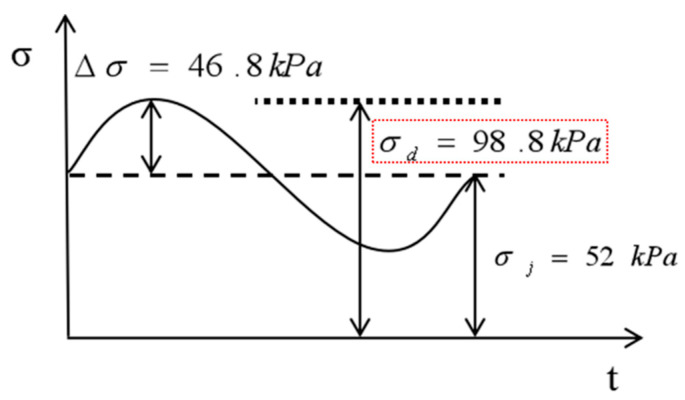
Stress–time curve.

As shown in Formula (4), when the train passes at high speeds, the stress generated by the subgrade bed surface consists of two parts: the static stress corresponding to the self-weight of the line superstructure ($\sigma_{j}$), and the additional dynamic stress corresponding to the load of the moving train, ∆σ. From these calculations, the static stress and the additional dynamic stress are $\sigma_{j}=52\;\mathrm{kN}\;\mathrm{and}\;\;∆\sigma =46.8\;\mathrm{kN}$. The design dynamic stress amplitude of the subgrade is $\sigma_{d}=98.8\;\mathrm{kN}$, and its change curve with time is shown in Figure 9.

#### 4.4.2. Excitation Frequency

1. Static load determination

In the field excitation test, the concrete loading plate base selected a convex base, a bottom area of 1.8 m × 1.8 m, a thickness of 0.1 m, an upper size of 2.5 m × 2.5 m, a thickness of 0.3m, a capacity of 23 kN/m^3^, a self-weight of the excitation equipment of 175 kN, and the calculated static stress generated by the excitation equipment and the concrete loading plate base was 69.62 kPa.

2. Dynamic load determination

In this experimental system, when the two eccentric blocks rotate at the same speed in the reverse direction, the excitation force is synthesized in the vertical direction, and the calculation formula is as follows:

(5) $$F_{\max}=m_{0}e{\left(2\pi f\right)}^{2}$$

where *m*_0_ is the total mass of the eccentric block; *e* is the eccentric distance of the eccentric block assembly (fixed); and *f* is the excitation frequency.

From Formula (5), it is known that, when the mass of the eccentric block is certain, the excitation frequency can be changed, and then the magnitude of the excitation force can be adjusted. Therefore, in the test, by adjusting the excitation frequency and combining it with the feedback value of the dynamic soil pressure box on the bed surface, the applied dynamic stress meets the design requirements. It was found that when the excitation frequency was 15 Hz, the measured dynamic stress amplitude on the bed surface was about 40 kPa. After the superposition of static stress and additional dynamic stress, the dynamic stress was 109.62 kPa, which is greater than the required stress amplitude, and therefore the loading frequency can be 15 Hz.

#### 4.4.3. The Number of Excitations and Frequency of Observations

Drawing on the existing literature for high-speed railroad excitation test research results [28,29,30,31], it was found that a number of excitations of 1.5 to 2 million times can better meet the simulation of the high-speed railroad roadbed surface loading requirements. In this paper, the object of study is the same as the high-speed railroad roadbed structure, so the number of excitations can be taken as 2 million times in the test, drawing on previous research. The field measurements found that the number of excitations can better meet the test requirements. According to a large amount of practical experience [32], in the early stage of excitation, the dynamic and static deformation characteristics of the bed change is relatively large, and so the field test should increase the collection density. Data were collected once every 10 min for the first 100,000 vibrations, and once every half hour thereafter.

## 5. Test Results and Analysis

To study the antidrainage performance of the new antidrainage structure and the dynamic characteristics and long-term stability of the diatomaceous earth subgrade, a typical test section was selected, and a field excitation test was designed. The soil moisture variation and the dynamic response of the antidrainage subgrade structure were determined to evaluate the antidrainage effect of the new antidrainage layer, and the propagation law of the dynamic response within the subgrade was studied.

### 5.1. Water Content Analysis at the Bottom of the Subgrade Bed

#### 5.1.1. Monitoring of the Water Content of Natural Foundation Subgrade Beds during Construction

Rainfall in the Shengzhou area is abundant from August to November, which has a significant impact on the construction unit’s filling operations; at the same time, diatomaceous earth disintegrates in the presence of water, and there is bound to be a significant impact on the strength and settlement of the natural foundation of diatomaceous earth in rainy areas. Under these conditions, the natural foundation of diatomaceous earth must be drained well, so a new antidrainage subgrade structure was designed for the diatomaceous earth area in the test section. The water content at the bottom of the subgrade bed was monitored during construction, and the performance of the new antidrainage subgrade structure was analyzed under natural rainfall conditions.

**Figure 10:**
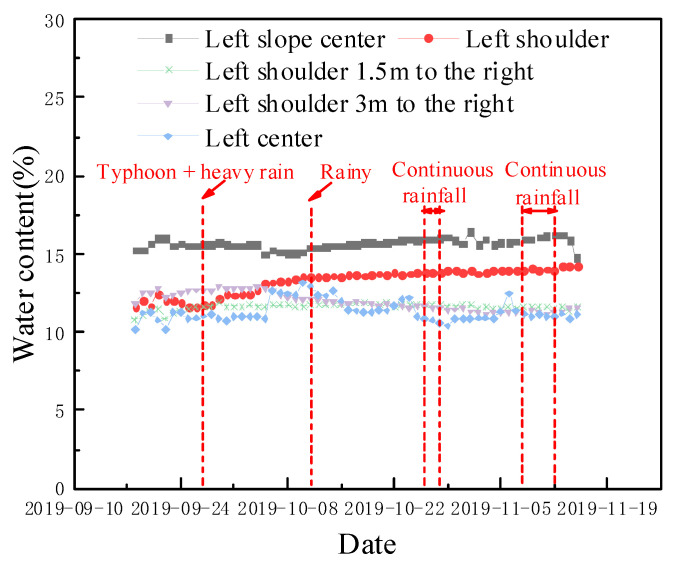
Time course curve for water content at the bottom of the subgrade bed during construction.

The change curve for the moisture level, which was determined by a meter located at the bottom of the subgrade bed, is shown in Figure 10. The moisture meter extracted data from 17:00 to 18:00 every day. The subgrade bed humidity level remained stable during the filling period and fluctuated within a sensor test error of ±2%. The new antidrainage layer effectively prevented the infiltration of rainwater in the rainy environment, thus reducing the adverse effects of rainwater on the natural foundation of diatomaceous earth.

#### 5.1.2. Monitoring of Subgrade Bed Water Content during Excitation Tests

**Figure 11:**
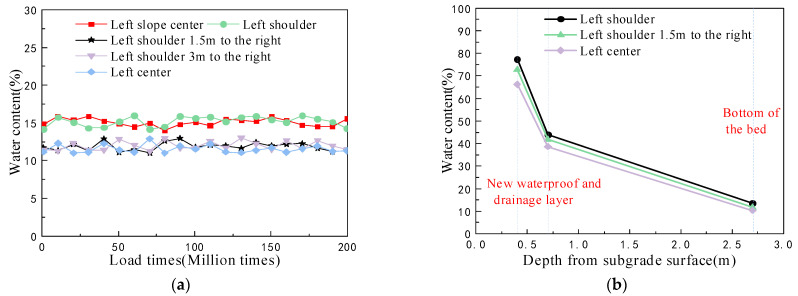
Variations of water content during the excitation test: (**a**) change in the water content at the bottom of the bed; and (**b**) change in the water content in the subgrade as a function of depth.

The excellent performance of the new antidrainage structure of diatomaceous earth was analyzed in the previous section, and the purpose of determining the water content in the bottom of the subgrade bed during the excitation test, as well as the variation in the water content with depth after the end of the excitation test, was to verify the rationality of the antidrainage structure under the most unfavorable working conditions. This included the coupling of a dynamic load and the immersion and protection of the lower diatomaceous earth foundation. The long-term stability characteristics of the antidrainage structure of the subgrade bed were examined, and the test results are shown in Figure 11.

1. Compared with the water content during the construction period, it can be seen that, during the excitation test, the water content at the center of the left slope of the subgrade and the left shoulder position was about 15%, and the water content at the rest of the positions was about 12%. During the loading process, the water content fluctuated very little in each test part of the substrate, which shows that the cyclic dynamic loading has little effect on the performance of the new drainage prevention structure in the substrate, and that the durability of the drainage prevention layer is good;
2. After the excitation test, the water content in the surface layer of the bed was much higher than the water content in the bottom layer of the bed below the new antidrainage layer, indicating that the antidrainage structure designed in the bed served as a good water barrier and that it effectively blocked the water in the upper section, and prevented rainwater from seeping down to the diatomaceous earth foundation [33]; this avoided the disintegration and deterioration of the diatomaceous earth and subsequent problems.

### 5.2. Dynamic Stress Analysis

#### 5.2.1. Analysis of the Dynamic Stress Changes Based on the Number of Vibrations

**Figure 12:**
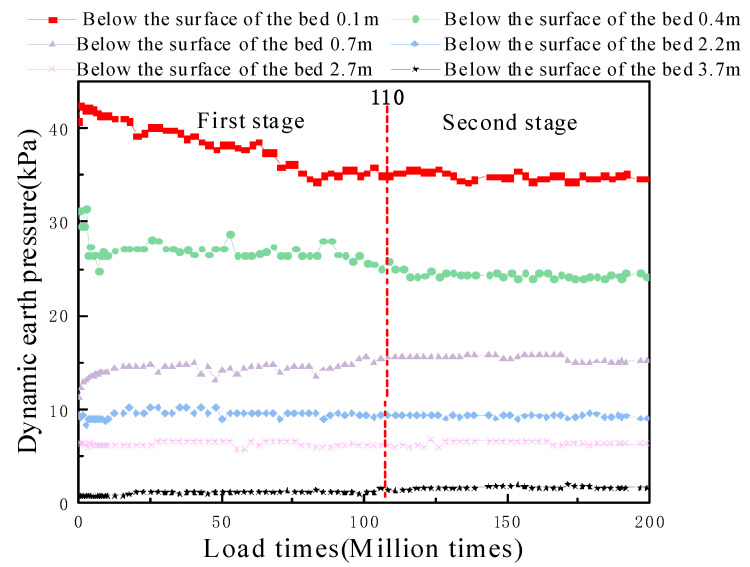
Plots of soil pressure with the number of vibrations—center depth of the left line track.

The magnitude of the dynamic stress directly reflects the impact of the train on the subgrade structure [34,35], and the dynamic stress changes, based on the number of vibrations, is shown in Figure 12. At the beginning of the excitation period, the fluctuations at the surface layer of the subgrade bed were more violent, mainly because of the graded gravel at 0~0.4 m in the subgrade bed. This phenomenon is similar to that obtained by Wang [36]: with increases in the excitation number for the submerged environment, the internal particles of the graded gravel layer adjusted until they reached a stable state. Fluctuations in the dynamic load at the location of the new antidrainage layer were relatively small and basically remained stable with the number of vibrations, which indicated that the new antidrainage layer had high stability under fatigue loading. The additional value of the dynamic stress at the base fluctuated from 5.7 to 6.7 kPa, and the average value after stabilization was 6.37 kPa. The dynamic stress at the foundation was small, and the average value of dynamic stress measured at 1 m below the surface of the foundation was 1.71 kPa. This indicates that the reaction coefficient of the diatomaceous soil foundation under excitation load is very small, and that the lower diatomaceous soil is free from dynamic stresses [37].

#### 5.2.2. Distribution Law of Dynamic Stresses along the Depth Direction

**Figure 13:**
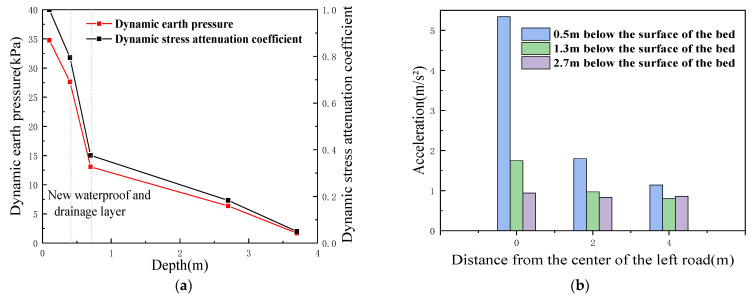
Analysis of dynamic stress test results: (**a**) variation law of dynamic stress with depth; and (**b**) distribution law of dynamic stress along the transverse direction.

By taking the center subgrade bed surface of the left line as the zero point, and the average value of the dynamic stress after stabilization as a representative value [38], the dynamic stress decay curve as a function of depth was plotted, as shown in Figure 13a. Because of the consumption of vibration energy and the damping effect of the bed fill, the dynamic stress of the bed structure gradually decayed from the top surface of the bed along with the increasing depth, and this phenomenon is similar to that obtained by Shang [39]. The dynamic stress decayed faster within the surface layer of the bed because the surface layer of the bed was small-grained graded gravel, which provided a good dispersion of the dynamic stress. The dynamic stress decayed by approximately 20% after 0.4 m of bed fill. The attenuation of the dynamic stress mainly occurred to a depth of 2 m under the test conditions. The new antidrainage layer produced an obvious attenuation of the dynamic stress, and the dynamic stress was reduced from 27.64 kPa to 13.06 kPa. This indicates that the new antidrainage layer has a certain energy dissipation and protection effect on the lower foundation, which is involved in vibration and which promotes the attenuation of the dynamic soil pressure [40], which is beneficial to the dynamic stability of the diatomaceous earth roadbed structure under the action of dynamic train loads.

#### 5.2.3. Dynamic Stress Distribution Law along the Transverse Side of the Subgrade Bed

Figure 13b shows the distribution of dynamic stresses along the transverse direction of the subgrade bed in a waterlogged environment. It can be seen that, in each depth direction below the surface of the bed, the dynamic stress is larger in the position close to the centerline of the track relative to other positions, and that with the increase in the horizontal distance, the dynamic stress decays gradually along the transverse direction; a similar law was obtained by Wang [41]. At the same time, this paper finds that, within 2 m from the centerline of the track in the transverse direction, the dynamic stress basically decays to 0, i.e., it is almost unaffected beyond 2 m from the centerline of the line. The decay rate is not uniform in all depth directions, and the deeper the depth, the slower the decay rate under the influence of dynamic stress.

### 5.3. Analysis of Vibration Acceleration

#### 5.3.1. Analysis of Vibration Acceleration with the Number of Vibrations

**Figure 14:**
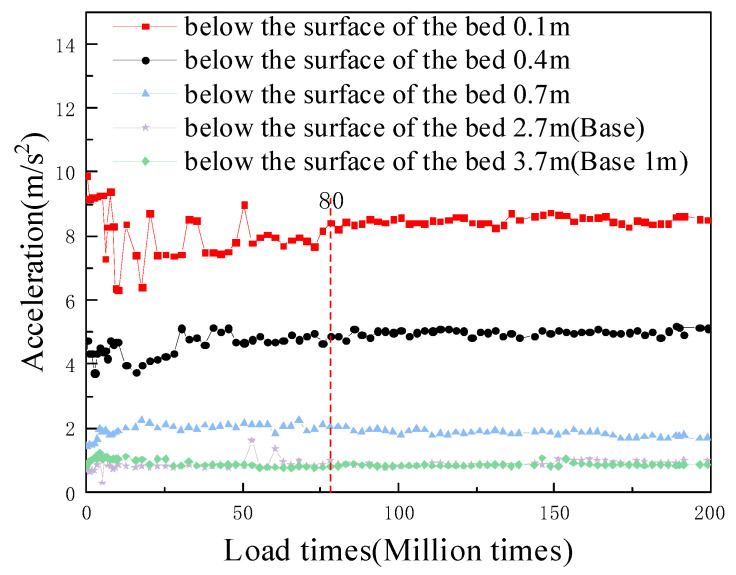
Variation law for vibrational acceleration with the number of vibrations.

A curve showing the vibration acceleration with the number of vibrations is presented in Figure 14. At the beginning of the test, the acceleration of the surface layer of the subgrade bed was more volatile, mainly because the sensor and the filler were in the coupling adjustment stage; this resulted in sudden changes in acceleration, and the amplitude of the sudden changes decreased with the increasing depth. With increases in the number of vibrations, the acceleration tended to gradually stabilize, and when the cumulative number of loadings was approximately 800,000, the acceleration at each position was basically unchanged; this indicated that the filler gravel was compacted after cumulative loading with 800,000 cycles. The vibrational acceleration at the bottom of the foundation bed was stable with the number of vibrations and remained at approximately 0.94 m/s^2^. This result is close to Chen’s result of 0.905 m/s^2^, obtained at an excitation frequency of 15 HZ [42]. At the same time, during the excitation test, the vibration acceleration curve for the foundation at a position 1 m below the bottom of the foundation always remained stable.

#### 5.3.2. Distribution Law of Vibrational Acceleration with Depth

**Figure 15:**
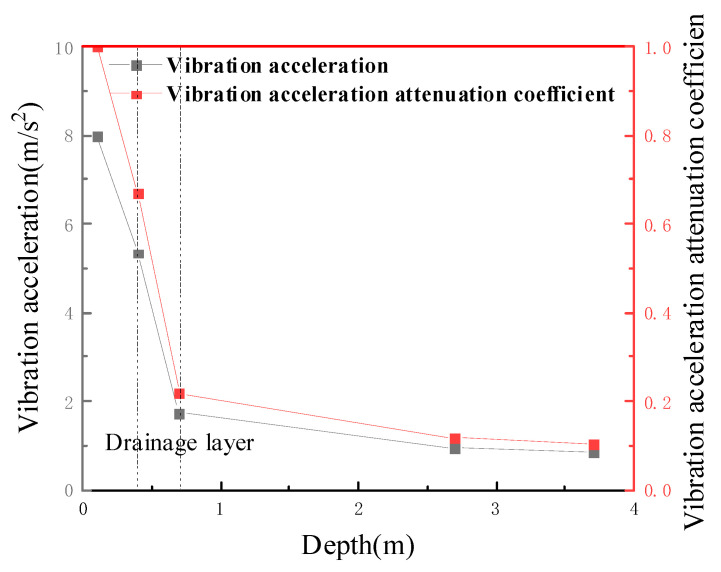
Variation law of vibrational acceleration with depth.

Taking the left line center subgrade surface as the zero point, and the average value of the vibrational acceleration after stabilization as a representative value, the vibrational acceleration decay with increasing depth was plotted, as shown in Figure 15. The acceleration of the bed structure gradually decays along with the depth, and the decay rate in the range of 0–0.7 m below the bed surface is significantly greater than that in the range of 0.7–2.7 m below the bed surface, mainly because of the damping effect of the bed surface fill and the new antidrainage layer, which makes the vibration energy effectively and substantially decayed in the range of 0–0.7 m [43]. The vibration acceleration was reduced from 5.34 m/s^2^ to 1.75 m/s^2^ after the energy dissipation effect of the new antidrainage layer, while the acceleration was attenuated to about 22% after the 0.7-m-thick bed surface fill and the new antidrainage layer. The attenuation of the vibrational acceleration decreased with increasing depth, according to a quadratic polynomial, and the vibrational acceleration decreased by approximately 90% from the surface of the subgrade bed to the contact surface at the bottom of the subgrade bed, and remained stable with increasing depths. This is mainly because the diatomaceous earth foundation in the test section is overlain by the stiff and structural basalt (hard shell layer), which ensures that the lower-lying diatomaceous earth is protected.

#### 5.3.3. Distribution Law of Vibrational Acceleration along the Transverse Direction of the Subgrade

**Figure 16:**
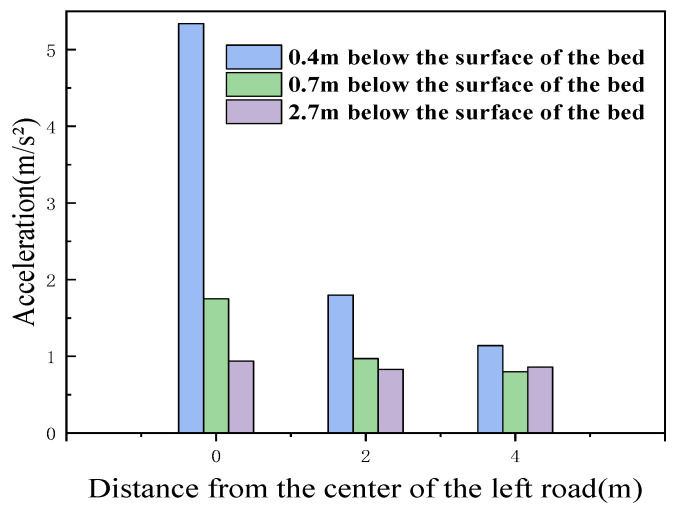
Distribution law for vibrational acceleration along the transverse direction.

Figure 16 shows the distribution law for the vibrational acceleration along the subgrade in a waterlogged environment. The vibration acceleration at the same depth of the roadbed structure is the largest at the location of the track centerline, and the farther away from the track centerline, the smaller the acceleration is, which is due to the upper load acting directly here, resulting in the largest influence of the upper load at the location of the track centerline; thus, this is where the vibration energy is the largest [44]. In each depth direction below the bed surface, the attenuation amplitude is larger in the transverse range of 2 m from the track centerline, and the attenuation amplitude is, relatively, much smaller, in the range of 2~4 m. The attenuation rate is not consistent in each depth direction, and the deeper the depth, the slower the attenuation rate is within the vibration influence range.

### 5.4. Analysis of the Cumulative Settlement of the Subgrade

**Figure 17:**
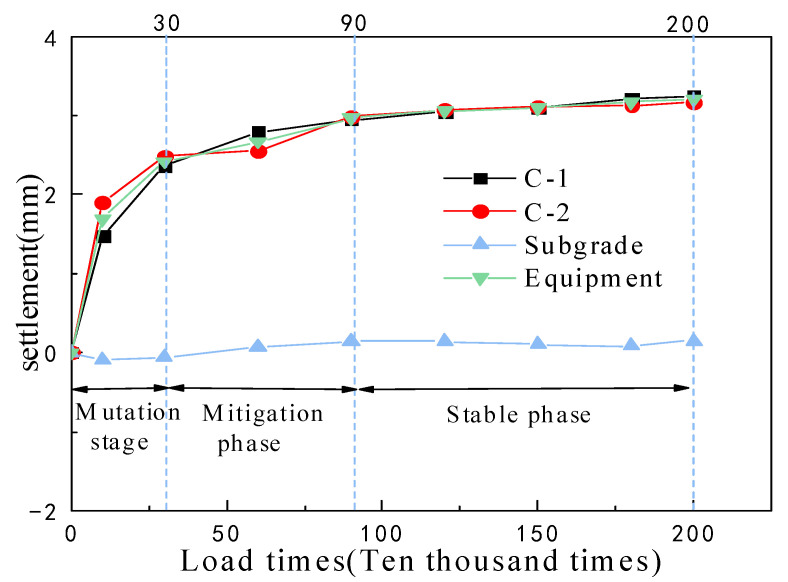
Variation law for cumulative settling with the number of excitations.

The diagonal points on the loading plate were selected as observation points (C-1, C-2) for determining the settling of the subgrade under fatigue loading, and a point on the subgrade surface near the loading plate was selected as the reference point for settling. The settling that occurred at each point during the excitation test is shown in Figure 17. The curve for the accumulated deformation of the subgrade surface with the number of loadings belonging to the decay curve and its deformation rate gradually slowed, and finally reached a stable state, indicating that the dynamic stress of the subgrade bed was less than its critical dynamic stress. The accumulated deformation of the subgrade bed was effectively controlled [27]. The number of vibrations in the sudden change phase and the slowdown phase in the figure correspond to the number of vibrations and the change in the dynamic stress; after 900,000 excitation cycles, the settling rate decreased and tended to stabilize. When the number of excitations reached 2 million, the accumulated plastic deformation of the subgrade under the loading plate was 3.26 mm, and the bed near the loading plate produced 0.15 mm of settling. Furthermore, the diatomaceous earth foundation produced 0.08 mm of settling, which indicates that the dynamic load had a significant influence on the settling of the bed. However, the postwork settling of the diatomaceous earth foundation caused by the dynamic load was negligible.

## 6. Conclusions

In this study, laboratory and field excitation tests were carried out at the test section of the diatomaceous earth subgrade for the new Hangtai railway, and the following conclusions were obtained:

1. The diatomaceous earth in the Shengzhou area is a regional geotechnical soil with very complex engineering properties. It has very poor hydrophysical properties and was easily disintegrated by water. The disintegration resistance index of the diatomaceous earth specimens ranged from 3.1% to 9.0%, with an average value of 6.05%, which means that they were weakly resistant to disintegration;
2. When diatomaceous earth was exposed to the natural environment, it was susceptible to disintegration and deterioration with frequent cycling between wet and dry, which led to problems such as softening and strength reduction, disintegration damage, penetration cracks, and other issues. This affected the strength and settling of the natural diatomaceous earth foundation, and the construction of projects in diatomaceous earth areas must be conducted with measures taken to prevent drainage;
3. For the first time, a new antidrainage subgrade structure was proposed for diatomaceous earth areas. During the vibration test, the attenuation of the water content in the new antidrainage layer range was about 30%, and the water contents of the test parts of the substrate fluctuated minimally, and were stable at 12% and 15% near the new waterproof structure under the coupling of the dynamic load, and the water immersion had good water impermeability and fatigue resistance. The dynamic stress and vibration acceleration through the capillary drainage layer ranged, respectively, from 27.64 kPa to 13.06 kPa, and from 5.34 m/s^2^ to 1.75 m/s^2^. The new drainage layer for the lower foundation has a certain energy dissipation and protection role;
4. Under the coupled effects of dynamic loading and water submersion, when cyclic loading reached 700,000 cycles, the dynamic stress and vibrational acceleration of the surface of the subgrade bed were stabilized at approximately 6.37 kPa and 0.94 m/s^2^, respectively. When the number of excitations reached 2 million, the settlement of the roadbed under the loading plate, the bed near the loading plate, and the diatomaceous earth foundation were 3.26 mm, 0.15 mm, and 0.08 mm, respectively. The settlement of the dynamic load on the bed was large, while the postwork settlement of the diatomaceous earth foundation caused by the dynamic load was negligible.

Because of the limited number of research years of the authors, the issues that can be further studied in the future are as follows:

1. Diatomaceous earth is a regional geotechnical soil, for which the test data accumulated thus far are limited, and the relevant test data are discrete. Therefore, a further improvement of diatomaceous earth test methods is needed;
2. In the future, on the basis of the roadbed settlement monitoring data, a prediction model conforming to the settlement law should be proposed, and the long-term stability of the diatomaceous earth roadbed should be studied and analyzed in depth;
3. The new Hang Shaotai high-speed railway will soon be operational and open to traffic. Thus, field dynamic tests can be carried out in the future to analyze the dynamic response characteristics of the roadbed structure when high-speed trains pass through the diatomaceous earth section.

## Figures and Tables

**Table 1:** Physical properties of diatomaceous earth.

Item	Classification	White Diatomaceous Earth	Black Diatomaceous Earth	Blue Diatomaceous Earth
Natural Density	Range	1.33~1.88	1.37~1.91	1.30~2.06
Dry Density	Range	0.64~1.58	0.82~1.50	0.67~1.62
Water content	Range	20.5~115.5%	27.5~102.2%	21.9~114.0%
	Average value	53.73%	68.02%	64.71%
Void ratio	Range	0.69~3.31	0.84~2.37	0.68~3.09
	Average value	1.47	1.71	1.85

## Data Availability

Not applicable.
